# Supplementary material for: Reinforcement learning when your life depends on it: A neuro-economic theory of learning
Source: PLoS Comput Biol. 2024 Oct 28;20(10):e1012554. doi: 10.1371/journal.pcbi.1012554 (PMC11542834; doi:10.1371/journal.pcbi.1012554)
Supplement: S1 Text — (PDF) [file pcbi.1012554.s001.pdf]

Supplementary Information
Reinforcement learning when your life depends on it: a neuro-economic theory of learning

Jiamu Jiang, Emilie Foyard, Mark C.W. van Rossum
University of Nottingham, Nottingham, UK

October 21, 2024

APPENDIX: Neuroeconomic Trade-off for learning

The optimal memory strategy maximizes the lifespan. The animal has to decide whether to invest energy in long term memory of the CS-US associate. We derive an expression for the change in lifetime given a small weight update Δw, which in turn leads to a small change in the hazards $\delta h(t)$. The lifetime of the not-learning animal l_{NL} can be approximated $l_{NL} = \sum_t \left[e^{-\sum_{t'} h^{NL}(t')} \right]$. For small changes in hazard the change in expected lifetime between a learning animal, denoted l, and a not learning (NL) animal l^{NL} can be Taylor expanded as

$$l - l^{NL} \approx -\sum_t \left[e^{-\sum_{t'} h^{NL}(t')} \sum_{t'} \delta h(t') \right]$$

Given ARM learning with a small weight change Δw, the temporary reduction in stimulus hazard is

$$\delta h_s(t) = |\Delta w| h_s^0 \frac{\partial P_-(w_-, w_+, \mu, t)}{\partial w_-} \exp(-t \log \gamma)$$

where γ is the decay rate (see main Eq. 8). Note that as the weight change decays, so does the hazard reduction.

For LTM learning, the expression is similar but the decay term is absent. Moreover, LTM learning at the same time increases starvation hazard. Assuming that the energy cost, $c_{LTM}|\Delta w|$, is small, it follows from main Eq.2 that

$$\delta h_M = c_{LTM} |\Delta w| h_M^{NL}$$

Pulling it all together, the difference in expected lifetime between ARM and LTM learning is in first order of $|\Delta w|$,

$$l^{ARM} - l^{LTM} \approx |\Delta w| \sum_t \left[e^{-\sum_{t'} h^{NL}(t')} \sum_{t'} \left\{ h_s^0 \frac{\partial P_-(w_-, w_+, \mu, t')}{\partial w_-} (1 - e^{-t' \log \gamma}) + c_{LTM} h_M^{NL} \right\} \right] \quad (1)$$

where as before h_s^0 denotes the stimulus hazard if it is approached.

Eq.1 describes the trade-off between ARM and LTM. When the lifetime difference is larger than zero, ARM learning should be chosen over LTM learning. Because learning decreases the probability of encountering the stimulus ($\partial P/\partial w < 0$), the first term in the curly brackets is negative, while the second term is strictly positive. While complex, the expression gives insight in when ARM memory is preferable to LTM, namely when the magnitude of the first term becomes smaller, and/or the magnitude of the second larger.

ARM is preferable when: 1) The stimulus hazard h_s^0 is small, 2) when the impact of the learning on the choice probability $\partial P/\partial w$ is small, e.g. after LTM has already been expressed, 3) the ARM decay γ is slow, or 4) the energy cost of LTM, c_{LTM} or the starvation hazard h_M^{NL} is high. Finally, the first exponential r.h.s term attenuates the benefit of long lasting memory, so that ARM is generally preferable when the expected lifetime is short.

Nevertheless, it would appear challenging for a fly to estimate the expected lifetime based on this expression to decide whether to use ARM or LTM memory, so instead we a looking for approximate heuristic algorithms that only rely on observables directly accessible by the organism and are close to optimal under various conditions.

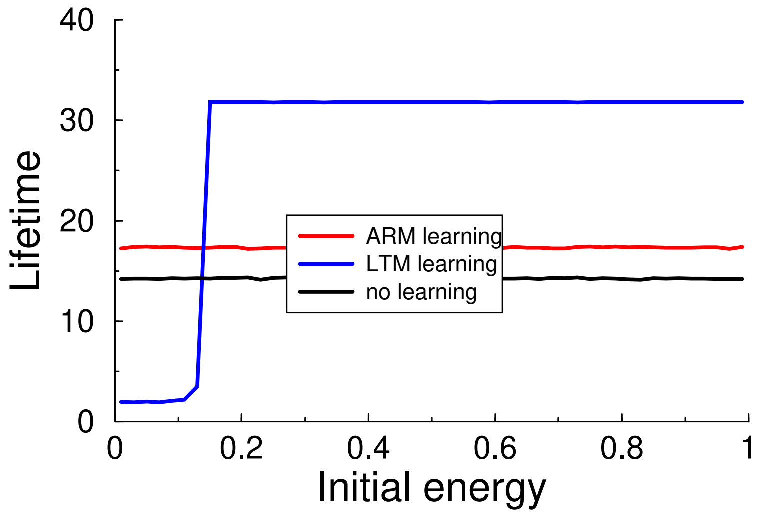

Supplementary Figure A: Life time as a function of the energy reserve using a sharp energy hazard. As main text Figure 4a, but the energy hazard is a step function: $h(E) = 1$ when $E \leq 0$, and the default level, $h(E) = 0.02$, otherwise. As before ARM learning (red curve) is always better than no learning (black line) and LTM (blue curve) is beneficial when the energy reserve is high, however the transition is sharper than in main text Fig. 4.

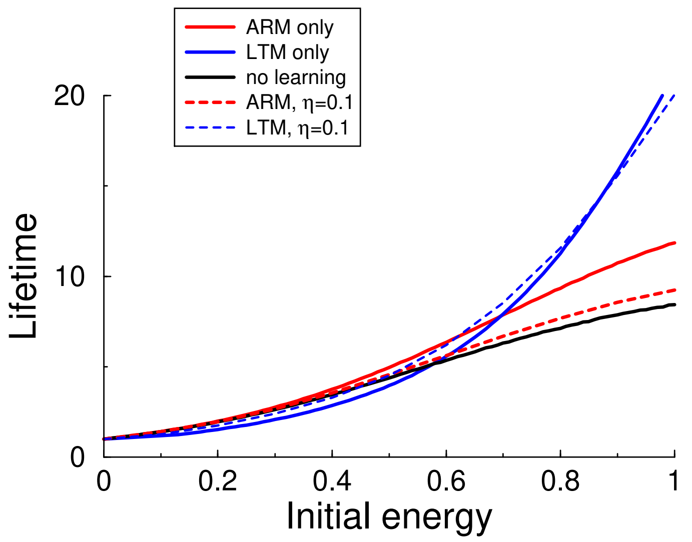

Supplementary Figure B: Effect of learning rate on lifetimes. As main text Figure 4a but also shown are the lifetimes when the learning rate of ARM (dashed red curve), and the learning rate of LTM is reduced to 0.1 (dashed blue curve). For comparison the curves for the original learning rate of 0.6 are shown as solid curves. A reduced ARM learning rate struggles to counteract the ARM decay, as a result the lifetime with ARM learning is reduced (red dashed curve). A reduced LTM learning rate slightly benefits lifetime when the energy reserve is low, but reduces it when the energy reserve is high (blue dashed).

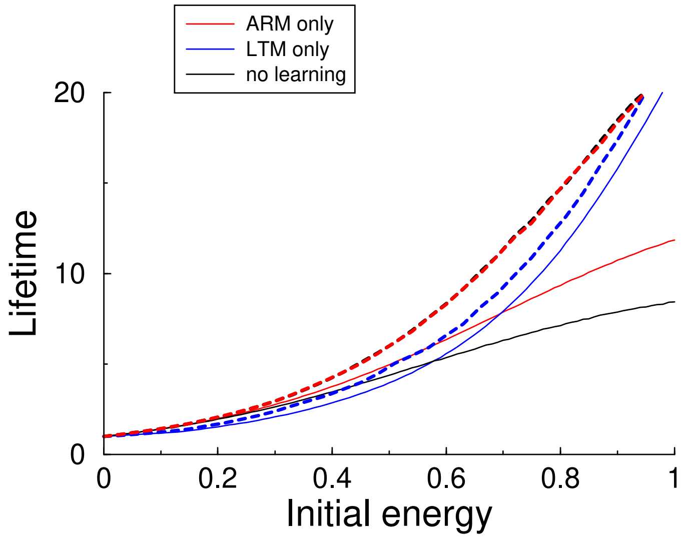

Supplementary Figure C: Effect of learning on lifetimes in case of a long inter stimulus interval. As main text Figure 4a when the interval is 5 days (dashed curves). For comparison the curves for the original 1 day interval are shown as solid curves. Overall lifetimes increase as for longer intervals there is less stimulus exposure. ARM learning decays so much that it carries no benefit over 'no learning' (overlapping dashed black and red curves). At rgw modeled hazard level, LTM learning always reduces lifetime in this case as the metabolic cost of learning outweighs avoiding the (sporadic) stimulus hazard.

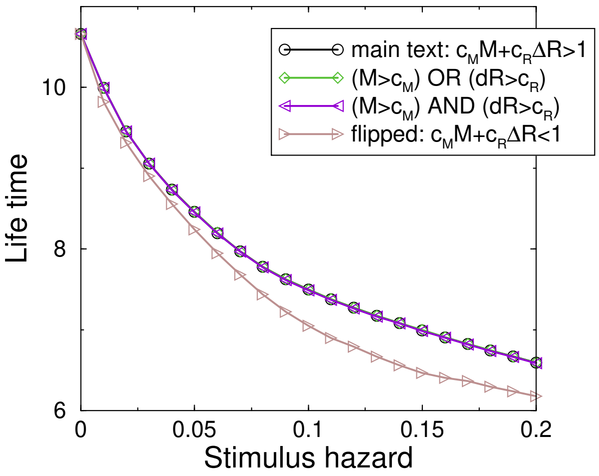

Supplementary Figure D: Effect of threshold algorithm on lifetime. As main text Figure 6c. Adaptive learning increases lifetime using either the algorithm from the text (black curve), or when applying LTM when energy reserve AND / OR reward prediction error exceeds a threshold (three overlapping curves). As a sanity check, when the threshold condition is reversed (LTM when energy reserve is low or reward prediction is low) the lifetime suffers. Parameters were optimized for each algorithm individually.

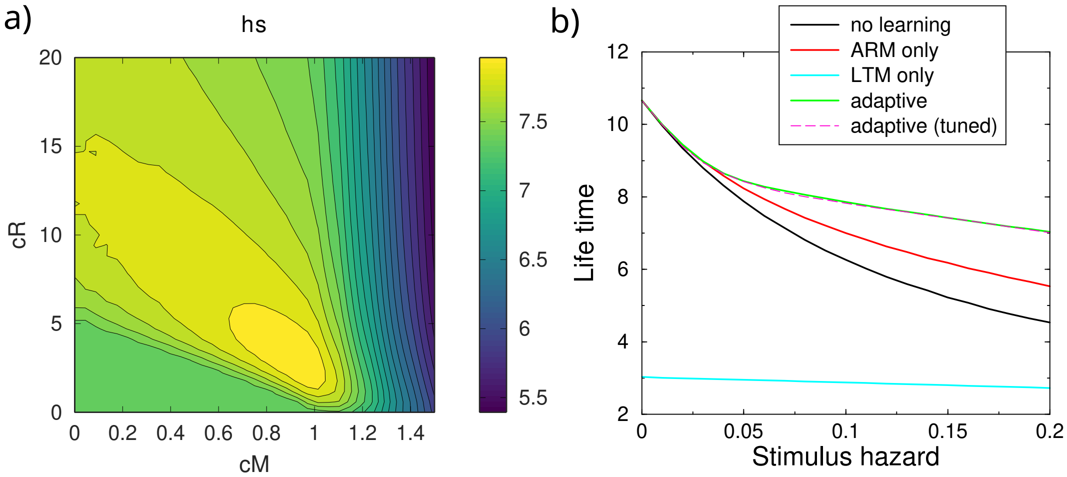

Supplementary Figure E: a) Life time for the adaptive learning model as a function of the threshold parameters. As main text Figure 6 but using the M_0 energy model.
b) Lifetime as function of the stimulus hazard. As for the M_1 energy model, the adaptive plasticity yields the longest life time.

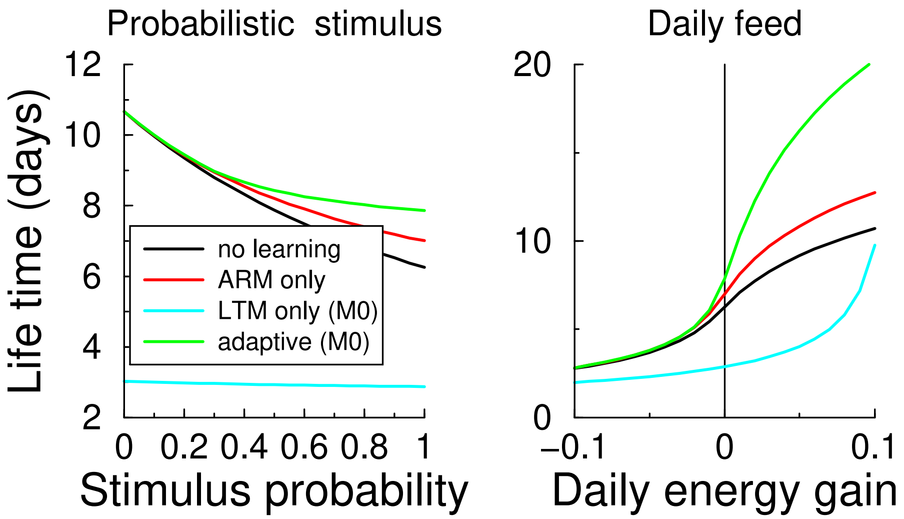

Supplementary Figure F: The life times for a probabilistic stimulus (left) and when there is additional intake or loss (right). As main text Figure 7, but for the M0 energy model. The adaptive algorithm robustly outperforms fixed strategies (Stimulus hazard 0.1).
