# Supplementary material for: Reinforcement learning when your life depends on it: A neuro-economic theory of learning
Source: PLoS Comput Biol. 2024 Oct 28;20(10):e1012554. doi: 10.1371/journal.pcbi.1012554 (PMC11542834; doi:10.1371/journal.pcbi.1012554)
Supplement: Attachment — Submitted filename: reply.pdf [file pcbi.1012554.s002.pdf]

Mark van Rossum
School of Psychology and
School of Mathematical Sciences
University of Nottingham
mark.vanrossum@nottingham.ac.uk

August 15, 2024

Dear Barbara/Dear Daniele

We are glad that the overall tone of the reviews was very positive and we like to thank the reviewers for their constructive comments. Thank you for giving us the opportunity to improve our manuscript. Most suggestions were adopted. Two major changes concern multiple reviewers:

Major Change 1 Appetitive association: Spurred on by the reviewers, we have further explored appetitive conditioning. We have investigated in particular larger rewards, so that the effects of learning are now much more obvious. See updated Fig.8

Major Change 2 Inter-stimulus timing: We have now included the effect of longer intervals between the conditioning stimuli. The main effect is that when the inter stimulus interval is longer, because of its decay, ARM plasticity becomes more similar to the 'no learning' condition, but the hazard rate also decreases. See updated main text Fig.8 (right) and Supp. Fig. 3 (for the case of appetitive conditioning).

Other changes and replies:

Reviewer #1:

1) I think the biggest question mark is how the model scales to multiple memories. Do the tradeoffs between LTM and ARM become more or less stark when there are multiple associations that the animal is confronted with? Does this depend on the time between stimulus presentations?

This is an interesting question from both a learning and an energetics point of view, as we now mention in the Discussion. Concerning the energy requirements, it would be very interesting to know how much energy it costs to learn two associations compared to one association as both cooperative and competitive effects could emerge, but we are not aware of experiments that have researched this matter. In the model the Kenyon cells are assumed to be sparsely coding and each additional association would simply be independent of the already present ones. Our energy measures would scale linearly in the number of associations, and the trade-off would not be altered. Should other odours active the same Kenyon cells, the animal would generalize to that odor. Thus this is mostly determined by the specific KC coding.

Regarding the role of the stimulus interval, see Major Change 2.

2) The variables in Fig. 2 should be defined in the caption.

Figure updated and caption corrected.

3) The conclusions of the section on appetitive conditioning seem relatively weak. This seems likely to stem from an assumption that flies re-encounter the same stimulus every day. If a rewarding stimulus is encountered only sporadically, how will things change?

We now include simulations where the reward is much larger, as well as simulations with a longer stimulus interval, hopefully clarifying this issue (see Major Change 1 and 2). We sincerely thank the reviewer for this comment, as it has made the results much clearer.

Reviewer #2:

...the principle of maximising lifetime in an RL context has been described in other domains...

We would be happy to include references regarding this as they would broaden the scope of the paper.

1) In the few cases where some parameters or hypotheses of the model were determined arbitrarily, it would be interesting to give insight on how robust the results would be with respect to deviations from these choices.
For example: how critical is the choice of an exponential law for the starvation hazard (eq.2)?
We have now included simulations where the starvation hazard is a sharp step function (see supplementary figure 1).

What if the ARM and LTM learning rates are not assumed to be equal?

We have now included simulation where either the ARM or the LTM learning rate are reduced (see supplementary figure 2).

How do the results vary (qualitatively or quantitatively) if the cost of LTM (cLTM) is varied?

This manipulation is now discussed in the text.

2) There are possibly some points of concern on Fig.3b, which seems in contradiction with the corresponding main text. The histogram on the figure indicates that the ARM-only condition gives a shorter lifetime than M1-LTM (given that there are more flies with longer lifetime in the M1-LTM condition), but the main text states the opposite (line 208). Also, it looks like the number of flies for the M0-LTM condition is larger than 10000 (the 4 bars are each >10^4). What is the explanation for that ? I might have misunderstood what is actually plotted, but if so I might not be the only one, and this figure would in that case deserve more explanation.

We thank the reviewer for spotting the issues with this figure which were caused by incorrect histogram normalization. We revised the figure.

3) In the final part, the authors adapt the model to appetitive memory. - A surprising result is that

appetitive memory seems to have very little beneficial effect, long-term memory in particular being almost always detrimental as compared to no learning. Given that 'real' animals do make appetitive LTM, it could be advisable that the authors provide a more developed discussion about which features, specific to appetitive memory, their model fails to capture – or erroneously captures. Related to the previous point, could it be a wrong estimation of the energy gain associated with reward ingestion compared to the actual memory cost? Or of the efficiency of appetitive LTM formation, which is known to form after a single trial in starved flies?

We sincerely thank the reviewer for drawing our attention to this. See Major Change 1. We extended our discussion of LTM formation under starvation.

Could the authors elaborate more on the absence of effect of learning (even at zero metabolic cost) on lifetime in the regime of strong negative daily energy intake? As the initial energy level, and the value of the daily energy intake are strong determinant of the lifetime in the 'no learning' condition, this seems very counter-intuitive as learning would tend to increase the daily energy intake. I don't see what in the model could be at the origin of this (lack of) effect.

We hope that the revised figure clarifies this. For intermediate negative intake, learning does help, but in the extreme case of large daily consumption the energy can not be compensated by the reward and the animal is bound for starvation regardless. Clarified in manuscript.

Also, could the authors detail on which basis it is speculated that ARM and LTM also exist in appetitive learning in flies?

This was a bit sloppy. For the model's purposes we meant any transient, decaying memory i.e. STM. Text updated.

On Figure 8b, is the 'daily energy gain' including the amount of energy received by eating the reward (in that case in it different from the aversive case of Figure 7) or is it a fixed amount that is received independently of the behavioral choices?

This is a fixed amount in addition to possible energetic rewards. Now clarified.

In addition to these concerns, minor clarifications are needed: In Figure 7b, is the stimulus occurrence probabilistic as well (as on the left panel), or is it fixed as in the previous cases?

It is fixed to one. Now stated in the caption.

L. 129: from what I understand, the R- parameter remains equal to zero at all times, and so does its expectation value "R-bar". Do authors confirm, and why is this parameter included in the model, then?

This is correct. It is included in the model for generality only and could be omitted. It is however standard in computational RL models. We now state this reasoning. (see also reply to Reviewer 3).

(Minor corrections were all adopted unless noted here.)

l.61 : It is surprising that the P(t) formula yields P=0 at all time for h=0 (i.e. no hazard). Do the authors confirm ?

Formally, this equation is not valid when $h = 0$, but is only valid in the limit of $h \to 0$, in which case P becomes a very stretched exponential. When $h = 0$, the flies never die. We have added the condition $h > 0$ to the text.

Reviewer #3:

There are some important modelling assumptions, described in more detail below, that would benefit from further clarification and discussion, as there are several discrepancies between the assumptions and experimental evidence. (...)

2) that ARM or LTM formation depend only on energy reserves, and not on each other;

Strictly speaking LTM and ARM formation are very strongly dependent on each other: either ARM or LTM is expressed (see Isabel et al. reference). So they are fully anti-correlated.

(...) ARM and LTM have the same learning rate.

We have now included simulation that show what happens when the learning rates differ (supplement figure 2). Of course, the model is only a caricature of the full dynamics of learning and for instance doesn't include delayed expression of LTM, as is now further emphasized in both Methods and Discussion.

1) The formation of ARM and LTM depend on the conditioning protocol. LTM is only acquired when spaced training protocols are used, whereas ARM is acquired after massed training. This is an important factor for how these two types of memory are formed, and it goes beyond energy reserves alone. See [Tully et al. (1994), Cell 79, 35-47] and [Isabel et al. (2004), Science 304, 1024-1027]. Currently, memory induction in the model presented here is the same for both ARM and LTM, and only depends on the energy reserve. Is it important that LTM and ARM are specifically modelled? Could ARM and short term memory (STM) be modelled instead, or ARM and medium term memory (MTM), or STM and MTM? Unfortunately, I do not know whether a similar difference in energy requirements hold for these other memory types. This would circumvent the difference with respect to spaced training, although ARM typically results from massed training of multiple associative exposures, whereas STM results from a single exposure, or at most a few exposures, but at least this would entail less of a difference in the conditioning protocol.

While the reviewer is correct that ARM and LTM induction depend on the stimulation protocol, it also depends on the energy reserve, as was shown in the Placais and Preat paper. Indeed there the *same* protocol, resulted in either ARM or LTM depending on energy level. Our paper focuses on this difference only. We have now emphasized this in the text.

In that sense, our use of the term ARM is a bit broad and could be interpreted as 'any metabolically inexpensive decaying memory'. We now state this more clearly. We are not aware of studies that have

picked the metabolic requirements of different forms of decaying memory further apart.

As we now more extensively discuss, it is tempting to speculate that the fact that the protocol dependence (LTM being induced by spaced stimulation), has a functional benefit. Such interpretations, albeit without specific metabolic consideration, but rather as a capacity constraint, have been put forward in the context of human psychology and memory spacing laws for instance in the cited paper by Anderson and Schooler. This goes outside of the scope of the paper, however; we are currently analyzing this effect.

2) More recently, [Jacob and Waddell (2020), Neuron 106, 977-991] demonstrate that, in differential conditioning, LTM is acquired after spaced training, but is expressed as a bias to approach the CS-, and is encoded in a smaller avoidance MBON response to the CS- than to the CS+. This is in contrast to what one might expect: a bias to avoid the CS+, which would be encoded by a smaller approach MBON response to the CS+, as has been modelled in this manuscript. A parallel, decaying ARM memory, specific to the CS+, is encoded in the approach MBONs, but not in the avoidance MBONs. Thus, the formation of LTM in flies after spaced aversive conditioning involves a transition from a CS+ avoidance ARM to a CS- approach LTM. The paradigm used in this manuscript, however, is slightly different, in that there is no CS-, and it is not clear in Jacob and Waddell (2020) whether their results extend to stimuli that were not experienced during conditioning, i.e. whether all stimuli gain an approach LTM as for the CS-. Nevertheless, these results suggest that ARM and LTM are not mutually exclusive per se, but that both may result from plasticity at the inputs to different MBONs, which persist for different durations. More important, these results suggest that LTM formation entails ARM formation and decay, which is also supported by Isabel et al. (2004). Consequently, it is also important to discuss the suitability of using the same learning rate for ARM and LTM, as LTM forms after ARM. This needs to be addressed in the Methodology and in the Discussion, as the current implementation of the model does not capture these important details, and may be misleading to the naive reader.

We have now added the effect of learning rate variations (suppl. fig. 2). However, as we now emphasize, the model only includes simple first order dynamics of plasticity. It excludes phenomena such as delayed expression, or transformation from ARM into LTM.

3) Spaced training is addressed briefly in the Discussion, where it's proposed that the fly should switch from ARM to LTM when the interval between conditioning exposures is increased. This is an appealing hypothesis. However, in experiments, the spacing interval is typically on the order of 15mins, which seems too short for the decay time of ARM, which is on the order of hours to days. A simulated experiment, with a smaller time step, to demonstrate how LTM has an advantage over ARM under spaced training protocols, would be an excellent addition to this manuscript, though I would not require it.

We now include simulations with longer stimulus intervals (see Major change 1). We note that there might a non-linear relation between optimal decay of the memory pathway and stimulus interval (see our discussion of spacing above).

Minor comments:

1) Fig. 1C would benefit from additional explanation. I understand that stimuli further in the future have less impact on the expected reinforcements, as would be the case for discounting, but labelling the vertical axis as "Lifetime reduction" confuses me, because it includes that fact that the animal must live longer in order to see the stimulus further in the future. Some other confusing aspects that may help to clear this up include: i) does the vertical axis convey the amount by which the lifetime is reduced (larger number implies greater reduction, with no upper limit), or the fraction of the lifetime that would be lived if the stimulus were not encountered (smaller number implies greater reduction)? ii) Does the horizontal axis convey the duration over which the stimulus is experienced, or the time from now at which the stimulus will be next experienced? iii) is this plot meant to be the survival function over time, i.e. the cumulative product S(t) = exp(-ht), where S is the survival probability, h is the hazard rate, and t is time?

The figure in the main text shows the absolute (not relative) reduction of life-time in days. The raw life-time are shown here:

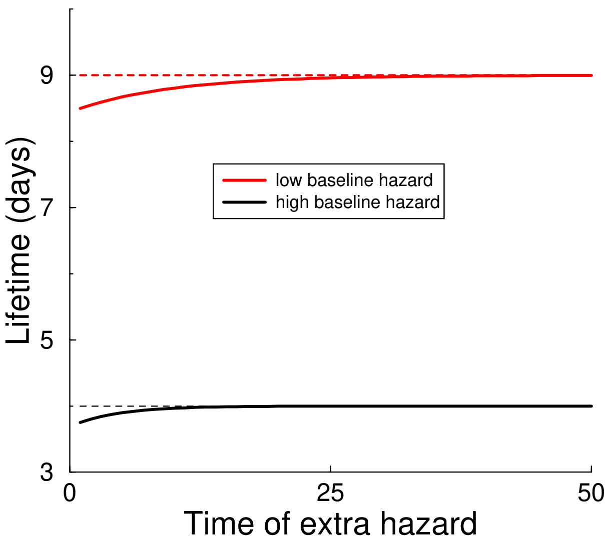

We now added units to the y-axis of the original figure ('days') that was erroneously ommited, which should remove confusion.

2) Line 73-75 – I'm finding it difficult to understand these sentences. If M(t) is positive valued, then h_M is always 0. This doesn't seem correct, unless I have misunderstood something. Could you please explain in more detail why you are using this new form for h_M, rather than that defined in Eq. 2.

We have now included a simulation for the steep threshold case, Supp. Fig. 1 (see also reply to reviewer 1). Otherwise Eq.2 is the one we use throughout. We incorrectly stated that the hazard would be zero when $M > 0$, it would need to be a small constant value instead (see also reply to reviewer 1).

4) Line 87-90 - As per my previous comment on Fig. 1c, am I correct in thinking that the lifetime reduction is a prediction? As such, it makes sense that stimuli further into the future have less of an effect on predictions of lifetime reduction. It would help if this predictive nature were made explicit. If

it is an absolute lifetime reduction, are we to interpret lifetime reduction as the integral of the lifetime reduction curve, up until the time at which the stimulus is presented?

We hope that the above comments have resolved this. It is a logic consequence of the hazard formulation.

7) Eqs. 6-7 – For those who wish to build upon this work, I think it would help to state that R-bar_ +/- could, in general, be cue specific, and depend on w_ +/-, even though only a single cue is modelled in this paradigm. If multiple cues were modelled, whereby the weights being updated depend on the sensory cue (Eq. 5), and R-bar_ +/- were literally a sliding window average over reinforcements for all cues, delta_w +/- would be larger on average, which would affect the optimal thresholds, c_M and c_R, for ARM vs LTM memory.

Excellent idea. Text extended.

8) Eqs. 6-7 – Following from point 8, if R-bar_ +/- does not depend on the actual reinforcement predictions encoded in the weights, then delta_w will be larger than needs be for LTM. If R-bar did depend on LTM weights, then R-bar wouldn't decay, and delta_w would be smaller on average for LTM formation, which in turn would affect the optimal c_M and c_R thresholds.. It would help to point this out explicitly, and to explain your choice model R-bar with a decay rate equal to that of ARM only.

Indeed! Methods text extended and reference to Bennett and Nowotny added.

11) Figure 3a - 1) the different line thicknesses in the bottom panel, for hazards, are difficult to distinguish. Can a different dimension of line style be used, e.g. solid, dashes, dots? In particular, for LTM, I cannot see three lines for the M1 model. If one is obscured by another, please state this in the caption.

There were some issues with Figure 3. It has now been revised.

12) Figure 3a - why does the stimulus hazard decrease at the beginning of the simulation, when it should be fixed at 0.2?

This is the stimulus *exposure* hazard, i.e. the hazard conditional on the probability of encountering it. Clarified.

14) Line 176-181 - The stimulus protocol, lasting 50 simulated days, seems at odds with the assumption made earlier that experiments are drastic such that age-related changes in the hazard can be ignored. That assumption makes sense when the model simulates an experiment lasting one day, but not a series of experiments lasting the lifetime of the fly. This choice of timescales needs more motivation.

The 50 days simulation is to make absolutely sure there are no survivors in the simulation, which would be ambivalent in the life time statistics. The typical lifetime is much shorter (some 10 days). Clarified.

18) Line 257-259 – please show the data that support these results.

We now include this as supplementary figure 6. We incorrectly stated originally that these model variants did not work as well, however, this was due to not correctly tuning the threshold parameters. In fact, they do as well (but not better).

20) Line 278-279 – Please show the data.

This is now included as supplementary figure 5.

21) The appendix in its current form is very difficult to follow, and would benefit from further explanation, suitable for a first year PhD student to follow. In particular, reintroducing symbols that were defined in the main text would be helpful. Some symbols have not been defined anywhere in the manuscript, e.g. lower case l, which I presume is lifetime, but had been defined using beforehand.

We have extended the explanations in the Appendix.

22) Line 409 - it is claimed that the equation shows that a transition from LTM to ARM is preferable late in learning, when dP/dw is small, but this is opposite to what happens in reality, which is a transition from ARM to LTM. Please provide further clarification in the Appendix text.

We hope that this is resolved by our above discussion of ARM and LTM and the inability of the model to have delayed expression.

23) Please provide instructions on Github to execute the code, including example commands.

We are working on this. (It is a pain when PhD students graduate!)

24) Code appears to be missing functions that plot all of the figures, which would greatly promote this work's reproducibility.
As above...

Yours sincerely, For all authors

Mark van Rossum
