# Supplementary material for: Reinforcement learning when your life depends on it: A neuro-economic theory of learning
Source: PLoS Comput Biol. 2024 Oct 28;20(10):e1012554. doi: 10.1371/journal.pcbi.1012554 (PMC11542834; doi:10.1371/journal.pcbi.1012554)
Supplement: Attachment — Submitted filename: reply2.pdf [file pcbi.1012554.s003.pdf]

Mark van Rossum
School of Psychology and
School of Mathematical Sciences
University of Nottingham
mark.vanrossum@nottingham.ac.uk

October 8, 2024

Dear Barbara/Dear Daniele

We like to thank the reviewers for helping to further improve the paper. We have corrected the manuscript as follows.

Reviewer #2:

- Figure 3b : the text in the legend box is duplicated.

Corrected.

- Figure 8a : No y-axis label ; legend box should not overlap with graphs. It would be better also to have separate x-axis labels.

Corrected.

Reviewer #3:

1) Line 79: "The result is a steeper transition..." - this appears to come before ARM to LTM transitions are discussed, begging the question: steeper than what?

Resolved by no longer mentioning ARM/LTM at this stage.

2) Line 150: The M1 energy model is referred to before being introduced. I suggest moving these two sentences about different learning rates to a later part of the paper, and replace it with a quick mention that different learning rates will be discussed later.

We refer forward now.

3) Fig. 1A: bottom panels need a legend to explain line thicknesses.

We presume this concerned Fig.3. Now fixed.

4) Line 303 and later: Could the word "consumption" be replaced with "expenditure" or something similar? I initially found this confusing, as "consumption" can have the same meaning as "intake", and suggested to me that energy was gained, e.g. from the consumption of food.

Replaced throughout.

5) It is interesting that the M1-energy model generally outperforms the M0-energy model when LTM is used exclusively, but when the adaptive threshold model is used, the M0-energy model outperforms the M1-energy model (comparing Fig. 6 with Supp. Fig. 5, and comparing Fig. 7 with Supp. Fig. 6). Are the authors able to comment on this and/or explain why, e.g. in the section "General threshold models"?

We don't have a good intuition for this. However, one should realize that the two models measure energy differently, so strictly speaking it would not be correct to say that one outperforms the other.

Yours sincerely, For all authors

Mark van Rossum
